# Promising Tools in Prostate Cancer Research: Selective Non-Steroidal Cytochrome P450 17A1 Inhibitors

**DOI:** 10.1038/srep29468

**Published:** 2016-07-12

**Authors:** Silvia Bonomo, Cecilie H. Hansen, Elyse M. Petrunak, Emily E. Scott, Bjarne Styrishave, Flemming Steen Jørgensen, Lars Olsen

**Affiliations:** 1 Department of Drug Design and Pharmacology, University of Copenhagen, Universitetsparken 2, DK-2100, Copenhagen Ø, Denmark; 2 Department of Pharmacy, University of Copenhagen, Universitetsparken 2, DK-2100 Copenhagen Ø, Denmark; 3 Department of Medicinal Chemistry, The University of Kansas, 1251 Wescoe Hall Dr., Lawrence, KS, 66045 USA

## Abstract

Cytochrome P450 17A1 (CYP17A1) is an important target in the treatment of prostate cancer because it produces androgens required for tumour growth. The FDA has approved only one CYP17A1 inhibitor, abiraterone, which contains a steroidal scaffold similar to the endogenous CYP17A1 substrates. Abiraterone is structurally similar to the substrates of other cytochrome P450 enzymes involved in steroidogenesis, and interference can pose a liability in terms of side effects. Using non-steroidal scaffolds is expected to enable the design of compounds that interact more selectively with CYP17A1. Therefore, we combined a structure-based virtual screening approach with density functional theory (DFT) calculations to suggest non-steroidal compounds selective for CYP17A1. *In vitro* assays demonstrated that two such compounds selectively inhibited CYP17A1 17α-hydroxylase and 17,20-lyase activities with IC_50_ values in the nanomolar range, without affinity for the major drug-metabolizing CYP2D6 and CYP3A4 enzymes and CYP21A2, with the latter result confirmed in human H295R cells.

Prostate cancer (PCa) is the second most common type of cancer in men and the fifth leading cause of death worldwide^1^. Several treatments have been developed against PCa, but drug resistance occurs rapidly, leading to a disease state known as castration-resistant prostate cancer (CRPC)^2^,^3^. In CRPC, androgens produced by the tumour and/or the adrenal gland drive disease progression. Thus, reduction or suppression of hormone levels in the cancer cells remains a key point in advanced stages of the disease.

Cytochrome P450 17A1 (CYP17A1) is a monooxygenase involved in the synthesis of steroidal hormones. CYP17A1 converts pregnenolone to dehydroepiandrosterone and progesterone to androstenedione by two subsequent reactions, the 17α-hydroxylase and 17,20-lyase reactions (cf. Fig. 1). The hydroxylase reaction generates intermediates in the biosynthesis of glucocorticoids, while both hydroxylase and lyase reactions are required for biosynthesis of androgens and oestrogens^4^. CYP17A1 is therefore a pivotal target in the treatment of hormone-dependent tumours such as prostate cancer^5^,^6^,^7^.

Several CYP17A1 inhibitors have been developed over the years, but only abiraterone (cf. Fig. 2) has been approved by the FDA for treating CRPC. Abiraterone consists of a steroidal scaffold with a pyridin-3-yl moiety in position 17 that inhibits CYP17A1 through coordination to the haem iron^8^. Oxygen binding to the haem iron is necessary for all CYP17A1 catalysis, so abiraterone binding is inhibitory. Together, the steroidal scaffold and the aromatic nitrogen-containing ring give abiraterone a promiscuous profile with affinity toward steroid receptors and other CYP enzymes, which likely contribute to the undesirable side effects observed in patients receiving abiraterone treatment^9^. Combinatorial synthesis programmes have been started by pharmaceutical companies to identify non-steroidal inhibitors and two such compounds, orteronel^10^ and VT-464^11^, have been evaluated in clinical trials.

Selective inhibition of CYP17A1 can be targeted by identification of non-steroidal compounds tailored to the three-dimensional structure of this particular enzyme by applying *in silico* screening of compound libraries. In this process, integration of structural information about the target protein in the virtual screening protocol typically increases the success rate for identifying hits with improved binding to the active site of the protein under investigation^12^,^13^,^14^. Despite the increasing number of cytochrome P450 X-ray structures, the presence of a haem cofactor makes these enzymes a challenging type of system from the computational chemistry point of view. This is because many inhibitors coordinate directly to the haem iron, *e.g.* with sp^2^-hybridized nitrogen atoms. Force field-based docking algorithms fail to properly describe this type of semi-covalent bond formation^15^,^16^. To overcome this problem, density functional theory (DFT) calculations were used to describe the nitrogen-iron interaction^17^ in combination with a haem-tailored structure-based virtual screening to suggest novel non-steroidal CYP17A1 inhibitors.

The ZINC^18^ and eMolecules^19^ databases were used as reservoirs of commercially available compounds. DFT calculations were used to select the N-containing heterocycles that most strongly coordinate to the ferric haem of CYP17A1 and to refine the docked binding mode. Compounds identified from the virtual screening were experimentally validated by determining their ability to bind to the CYP17A1 haem iron and to inhibit the catalytic activity of this enzyme *in vitro*. The most promising compounds were subsequently characterised for their possible disrupting activities on steroidogenesis in the human H295R adrenocorticoidal carcinoma cell line, a unique *in vitro* system that mimics the biosynthesis of androgens and oestrogens.

## Results

### Design of screening libraries

Some N-containing aromatic heterocycles can interact strongly with the ferric haem^20^,^21^ and the semi-covalent bond formed between the haem iron and aromatic nitrogen atom can only be described accurately by methods that explicitly consider electrons^17^. Density functional theory (DFT) methods have been successfully applied to describe this bond type and to calculate interaction energies between the haem iron and aromatic aza-rings^17^,^21^,^22^. The binding energy was calculated for the interaction of the most common sp^2^-hybridized N-containing rings with the haem using the DFT method according to the scheme shown in Fig. 3 and were in agreement with data previously published^21^. Imidazole, pyridine, 1,2,4- and 1,2,3-triazole, pyrazine, pyrimidine, pyrazole, oxazole, thiazole, isoxazole, 1,2,3,4- and 1,2,3,5-tetrazole and isothiazole exhibited the strongest interactions (Fig. 3). Compounds containing these fragments were then identified within the ZINC and eMolecules databases (see Methods and Fig. 4 for details).

### Virtual screening

A detailed overview of the workflow applied in this paper is presented in Fig. 4. The GOLD^23^ software was used for docking with the haem-tailored ChemScore function^24^ because it has been successfully applied in previous virtual screening studies^25^. A training set of 36 CYP17A1 ligands with known IC_50_ data^26^,^27^,^28^ was used to determine a cutoff for ChemScore to be able to distinguish likely inhibitors from non-inhibitors (see Figure S1, Supplementary Information, for 2D structures). The training set consisted of 23 known CYP17A1 inhibitors with IC_50_ values lower than 5000 nM and 13 non-inhibitors with IC_50_ values above 5000 nM. The distribution of ChemScore values is reported in Fig. 5 and clearly shows an enrichment of inhibitors when the ChemScore values were more negative than −50 kJ·mol^−1^. Therefore, binding modes with scores higher than this value were discarded, yielding 414 virtual hits for further analysis.

Boström and co-workers have shown that compounds with nanomolar affinity for their target proteins are characterised by a ligand strain, *i.e.* the energy difference between the most stable conformation in water and the one adopted in the binding pocket, less than 12.6 kJ mol^−1 ^29. Compounds with ligand strain higher than this value were therefore discarded. The resulting 51 protein-ligand complexes were optimized with a quantum mechanical/molecular mechanics (QM/MM) method to accurately model the Fe-N interaction within the CYP17A1 enzyme. For each compound, the refined binding mode was compared with the previously obtained docking pose in terms of distance between the haem iron and the sp^2^-hybridized nitrogen atom of the ligand. Compounds which did not retain the binding mode were discarded. The remaining 19 compounds (see Figure S3) were purchased and tested *in vitro* for binding to the CYP17A1 haem iron and inhibiting the enzyme 17α-hydroxylase and lyase activities.

### Experimental validation of the hits from the virtual screening

#### Binding to CYP17A1

Direct coordination of nitrogen-containing heterocycles to the haem iron in the active site of cytochrome P450 enzymes causes a shift of the Soret peak λ_max_ to higher wavelengths. Thus, UV-visible absorbance spectra were recorded as purified CYP17A1 was titrated with the 19 hit compounds. Addition of compounds **1–7** (cf. Fig. 2 and S3) yielded characteristic shifts in λ_max_ from 417 nm to as high as 427 nm, typical of direct coordination to the haem iron. Plots of the changes in absorbance as a function of concentration can be used to evaluate ligand binding affinity. Like abiraterone, compounds **1** and **2** yielded almost linear increases in absorbance, resulting in a generally poor fit even to the tight-binding equation (R^2^ of 0.809 and 0.903, respectively, to equation 2, cf. Figure S4). This result indicates that the apparent *K*_d_ values of compounds **1** and **2** are significantly lower than the CYP17A1 concentration (Table 1). Since CYP17A1 concentrations < 100 nM yields unacceptable signal:noise, the K_d_ values for **1**, **2**, **4**, and abiraterone can only be estimated as < 100 nM, although **4** showed weaker affinity. Compounds **3**, and **5** also demonstrated weaker affinity, permitting K_d_ values to be determined in the range of 150 - 400 nM (cf. Table S1). Compounds **6** and **7** have substantial intrinsic absorbance overlapping the range of the protein spectral change. Therefore the binding constants could not be determined for these latter two compounds.

#### CYP17A1 inhibition and selectivity against other CYP isoforms

The direct effects of these seven compounds on both CYP17A1 enzyme activities were evaluated using a purified protein system. First, the IC_50_ for the 17α-hydroxylase reaction was determined using progesterone as a substrate (cf. Table 1 and Table S1). Compounds **1** and **2** were the most potent inhibitors of this reaction with IC_50_ values of 230 and 130 nM, respectively. The IC_50_ values for inhibition of the 17,20-lyase reaction by **1** and **2** were 500 nM and 110 nM, respectively. Thus both compounds effectively inhibited human CYP17A1 catalysis with neither exhibiting marked selectivity for one CYP17A1-mediated reaction over the other (Table 1).

To investigate the selectivity of compounds **1** and **2**, their ability to bind to several other cytochrome P450 enzymes was also investigated. The steroidogenic CYP21A1 enzyme was evaluated since it shares the common substrate progesterone with CYP17A1. CYP3A4 and CYP2D6 were also evaluated as representative cytochrome P450 enzymes involved in drug metabolism (Table 1). Concentrations of 1 μM compound **1** and 0.1 μM compound **2** produced little or no spectral shift for CYP21A2, 2D6, or 3A4. Notably, these concentrations substantially exceed those that saturate CYP17A1, establishing that inhibitors **1** and **2** bind to these three isoforms with significantly weaker affinity than binding to CYP17A1, in contrast to abiraterone (sf. Table 1).

#### Human H295R cell assay

Compounds **1** and **2** were further evaluated in the human H295R cell line, a special *in vitro* system which contains all of the enzymes involved in the human steroidogenesis, including those required for androgen and oestrogen biosyntheses. Figure 6 and Table S2 show the effect of compounds **1** and **2** on steroidogenesis compared to abiraterone. Consistent with inhibition data, compounds **1** and **2** cause dose-dependent increases in the CYP17A1 substrates pregnenolone and progesterone, although higher concentrations were required compared to abiraterone. The rank order potency for inhibition of hydroxylase and 17,20-lyase is in agreement with the corresponding IC_50_ values reported above using purified CYP17A1.

This cellular assay was also used to evaluate inhibitor selectivity. At higher concentrations (30–3000 nM) abiraterone inhibited both CYP17A1 and CYP21A2, while at concentrations between 2.5 and 10 nM only CYP17A1 was affected. The IC_50_ for abiraterone inhibition of CYP21A2 activity was 240 nM. Compound **2** showed the same trend, reducing CYP21A2 activity at inhibitor concentrations above 1000 nM, although issues with solubility and cytotoxicity prevented detecting the full dose-response curve in CYP21A2. In contrast, for compound **1** IC_50_ values ranged from 300 to 1200 nM for progestagens and corticosteroids, and CYP21A2 inhibition was not observed (Table S2). The upper asymptotes for progesterone and corticosterone were not determined because of low solubility of compound **1**, which started to precipitate at concentrations around 10 μM. Thus, the 4 parameter log-logistic dose-response model was not significant for pregnenolone and 11-deoxycorticosterone because of the limited number of data points collected (cf. Table S2).

Overall, at low concentrations of **1** and **2** the pronounced and selective effects on CYP17A1 activity resulted in a decrease of androgen levels.

### Proposed binding modes in CYP17A1

The conformation of compound **1** identified from docking and subsequent QM/MM optimization is shown in Fig. 7a. The 2-(pyridine-3-yl)thiazole moiety occupies the same space as the 3-(cyclopent-1-en-1-yl)pyridine of abiraterone. The *m*-xylene part of **1** is predicted to form van der Waals interactions with Leu209 and Val482, while the thiazole system may make π-π interactions with Phe114. These contributions resulted in a ChemScore value of −50.4 kJ·mol^−1^. Compound **1** has limited flexibility with only two rotatable bonds. The dihedral angle around the pyridine-thiazole bond is 43° and the rotation of 50° between the planes formed by the *m*-xylene and thiazole rings result in ligand strain of 12.2 kJ·mol^−1^ which is close to the cutoff of 12.6 kJ·mol^−1^.

The predicted binding mode of compound **2** (cf. Fig. 7b) is characterised by the benzotriazole moiety coordinating the haem iron and a ligand strain of only 4.8 kJ·mol^−1^. The better ChemScore, −62.6 kJ·mol^−1^, compared to that for **1**, could be due to a predicted hydrogen bond between the cyano moiety and Arg239 and polar interactions of the amino group with Asp298. Moreover, non-polar contacts were predicted between the propyl chain and the phenyl ring with Val366, Ile317, and Val482.

Compound **2** also contains a pyridine ring capable of coordinating iron and, according to our DFT calculations, the pyridine coordinates iron more strongly than benzotriazole. To test the role of the pyridine ring of compound **2** in binding to the haem iron, eight commercially available analogues with other substituents in place of the pyridine (Figure S5) were also experimentally evaluated for coordination to the haem iron via UV-VIS spectrometry. Of these analogues, only the pyridine-3-yl counterpart (compound **2a**) demonstrated weak binding to CYP17A1. The rest of the series did not produce spectral shifts associated with CYP17A1 binding (data not shown). Thus, it seems unlikely that the benzotriazole ring of **2** coordinates the haem iron. A constrained docking, followed by the QM/MM refinement, was performed to evaluate the binding of **2** with the pyridine ring close to the haem iron (Fig. 7c). This binding mode had ChemScore and ligand strain values within the cutoffs set during the virtual screening (−52.9 kJ·mol^−1^ and 9.2 kJ·mol^−1^, respectively). In this predicted binding mode, the benzotriazole interacts with Arg239, while the neighboring pyridine ring made π-π interactions with Phe114, as also observed for the unconstrained docking.

## Discussion

**Figure 1:**
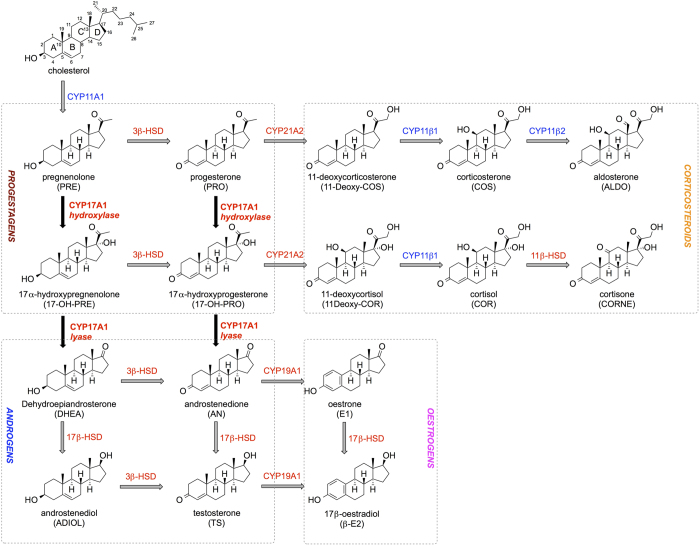
Summary of the steroidogenesis process. Enzymes coloured in blue are located in the mitochondrial membrane, while the red ones are present in the smooth endoplasmic reticulum. Reactions catalysed by CYP17A1 are reported in bold and black arrows. Abbreviations for each steroid are reported in brackets. Other abbreviations: HSD (hydroxysteroid dehydrogenase).

**Figure 2:**
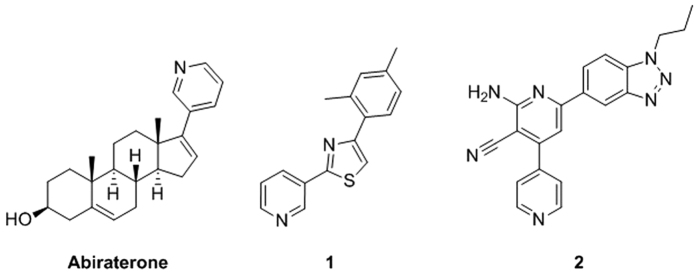
Structures of abiraterone and inhibitors identified in this study (1 and 2).

The link between prostate cancer and overproduction of androgen hormones is well established and has led to the development of androgen deprivation therapy (ADT). Unfortunately, cancer cells quickly acquire the ability to produce male hormones autonomously, resulting in resistance to ADT that is the hallmark of CRPC. CYP17A1 inhibition is an emerging strategy for treatment of CRPC by suppressing androgen production in all tissues (cf. Fig. 1). In this work, we aim at designing compounds that specifically inhibit CYP17A1. Another compound, abiraterone, effectively inhibits CYP17A1 (Table 1), but it also binds several drug metabolizing cytochrome P450 enzymes including CYP3A4 and CYP2D6 which may lead to adverse effects and toxicities^9^. Furthermore, abiraterone inhibits CYP21A2^30^ and CYP11B1^9^ which in the clinical setting, forces co-administration of prednisone to mitigate the resulting mineralocorticoid excess^30^. Besides the effects from the binding of abiraterone to CYP enzymes, it may have several other anticancer effects, involving binding to the androgen receptor, 3β‐hydroxysteroid dehydrogenase inhibition as well as decreasing the levels of heat shock protein 27, a cytoprotective agent involved in drug resistance issues, in androgen-insensitive prostate cancer cells^31^. Although these types of effects may be favourable, a selectively binding compound could reduce the risk of adverse side effects and can additionally be used as pharmacological tools in prostate cancer research.

Identification of inhibitors with improved selectivity can be facilitated by including structural information about the target protein in the early stages of drug discovery^12^. Many CYP inhibitors contain sp^2^-hybridized N atoms, which coordinate directly to the haem iron. This type of interaction is typically recognized by force field-based docking software via a point-charge-term which is most likely not representative of this bond. Therefore, Fe-N constraints are in some cases applied in screening compound libraries against cytochrome P450 enzymes^15^,^16^,^32^.

**Figure 3:**
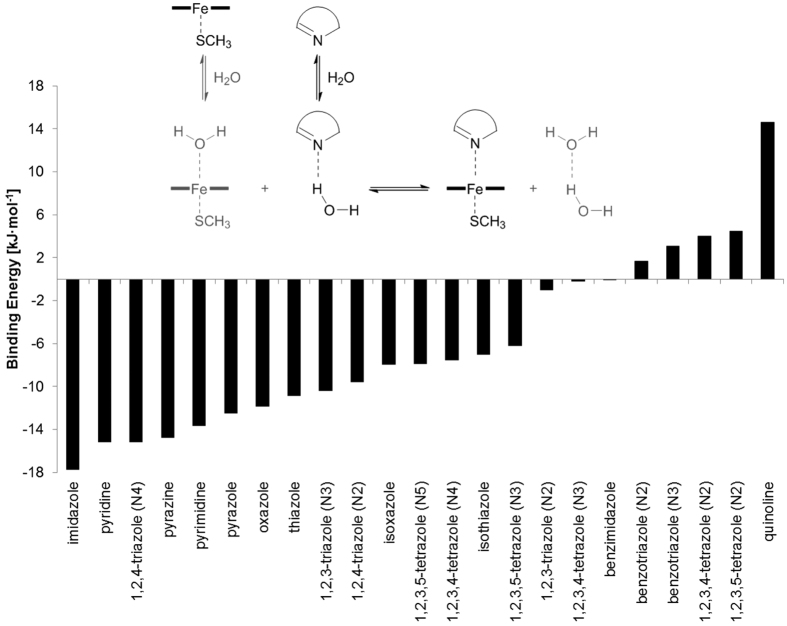
DFT-based binding energies of N-containing heterocycles. The scheme used by Leach and Kidley was applied^21^. The atom involved in the interaction with the haem iron is reported in brackets for rings with more than one N atom. The structures of the heterocycles are reported in Figure S1 of the Supplementary Information.

**Figure 4:**
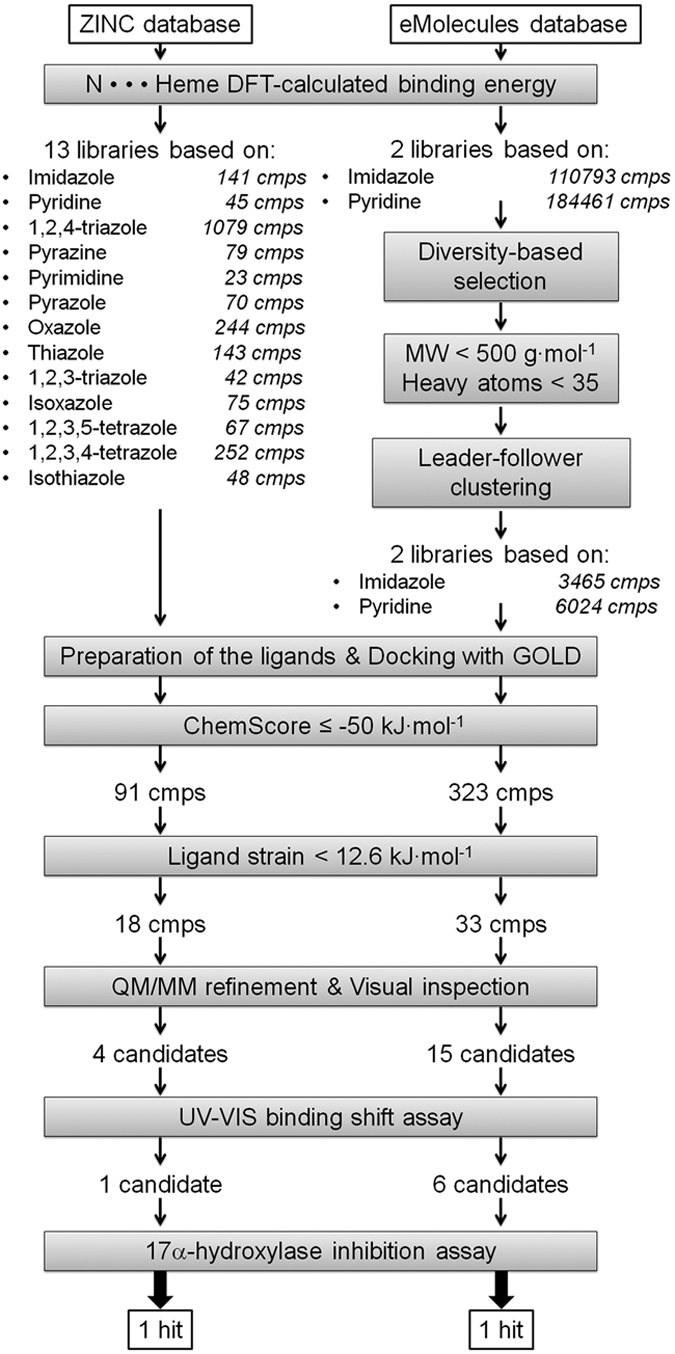
Overview of the virtual screening workflow.

**Figure 5:**
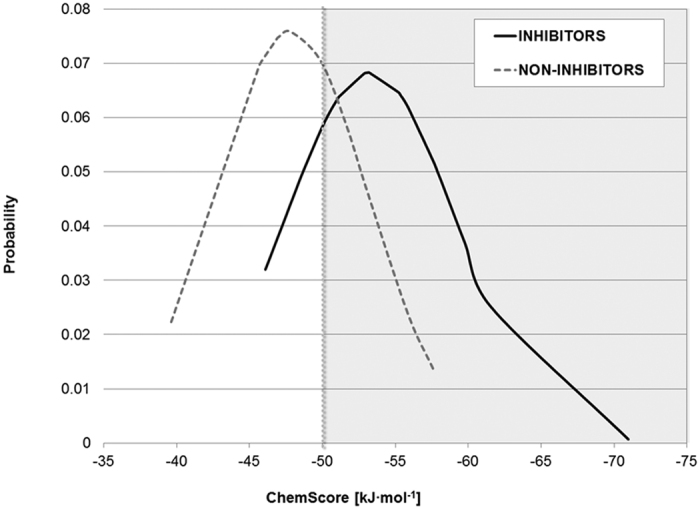
ChemScore distribution for a set of known inhibitors (black, solid line) and non-inhibitors (grey, dashed line). An enrichment of inhibitors starts with ChemScore values < −50 kJ·mol^−1^ (highlighted grey area).

In this work we discovered two non-steroidal CYP17A1 inhibitors that were selective over CYP3A4, CYP2D6, and CYP21A2 by combining a classical structure-based virtual screening approach using a haem-tailored docking protocol with a refinement of the Fe-N interaction by density functional theory (DFT) calculations. Imidazole and pyridine rings are the best haem-interacting heterocycles (Fig. 3) in agreement with previous data^21^. QM/MM refinement of the docking poses revealed that several of the imidazole-based compounds adopted Fe-N distances that were too long and so fewer compounds from this library were selected for biological testing.

The initial experimental studies using recombinant human CYP17A1 enzyme identified two inhibitors, **1** and **2**, with IC_50_ values of 230 and 130 nM, respectively (Table 1) for progesterone 17α-hydroxylase inhibition. The affinity of compound **2** is comparable to abiraterone, whereas compound **1** is 2–3 times less potent. Compounds **1** and **2** also inhibited 17α-hydroxypregnenolone 17,20-lyase activity, with IC_50_ values similar to those for the aforementioned progesterone hydroxylase reaction. Importantly, no binding was observed for compounds **1** and **2** to CYP3A4, CYP2D6 and CYP21A2, in contrast to abiraterone (cf. Table 1).

**Figure 6:**
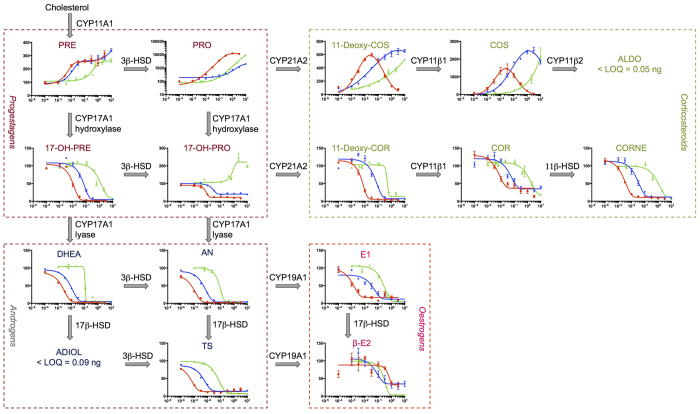
Effects of compound 1 (green triangles), 2 (blue diamonds) and abiraterone (red points) on the production of steroidal hormones in the H295R cell line. Data are reported as mean over 6–15 measurements ± standard error. The log of the inhibitor concentration, expressed in μM, is reported on the *x* axes while the *y* axes hosts the % of production of endogenous substrate for all the plots with the exception of PRO, where the *y* axes represents the log of % of production of PRO. Different scales have been adopted on the *y* axes to better show variations for each steroid. For the full label of hormones and enzymes refer to Fig. 1. Other abbreviations: LOQ (limit of quantification).

**Figure 7:**
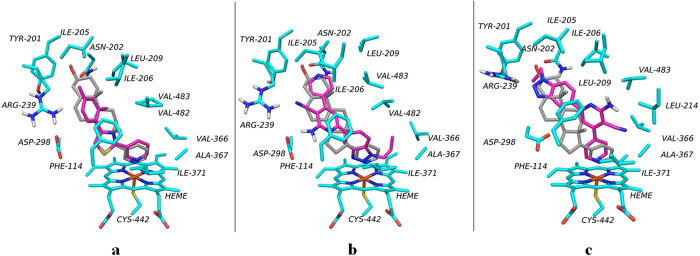
QM/MM-refined binding poses of **1** (**a**) and **2** (**b,c**), reported in magenta sticks, superimposed with the experimentally determined abiraterone position, shown in grey sticks. Amino acid side chains are coloured in cyan.

Compound **1** is smaller than abiraterone (cf. Fig. 7a) and is predicted to interact primarily with surrounding amino acids via π-π or van der Waals interactions. Compound **2** is comprised of a larger four-ring system, which may occupy the CYP17A1 pocket more completely. Initially, our work suggested a binding mode characterised by a hydrogen bond with Arg239, a polar interaction with Asp289, and coordination of the benzotriazole moiety to the haem iron (cf. Fig. 6b). However, direct coordination of the benzotriazole ring to the haem iron was not supported by our DFT calculations, which showed that the binding energy for the benzotriazole ring to the haem iron is less favourable than that of the pyridine ring (cf. Fig. 3). UV-VIS measurements on a series of analogues of **2** (cf. Figure S5) revealed that only compound **2a**, the pyridine-3-yl analogue of **2**, binds weakly to CYP17A1, which suggests that the benzotriazole moiety does not coordinate the haem iron.

Therefore, another binding mode of compound **2**, in agreement with both our DFT and experimental data, was identified. The pyridine ring coordinated the haem group while the benzotriazole moiety was predicted to hydrogen bond to Arg239. The ChemScore value and the ligand strain values of −52.9 kJ·mol^−1^ and 9.2 kJ·mol^−1^, respectively, were higher than those of the initial binding mode, but still better than those of compound **1.** Taken together, this data is consistent with **2** demonstrating higher potency for CYP17A1 inhibition than **1**.

The *in vitro* tests of compounds **1** and **2** in the H295R assay showed that they are able to penetrate the cell membrane and result in dose-dependent effects on steroidogenesis. Inhibition of 17α-hydroxypregnenolone 17,20-lyase activity by compounds **1** and **2** was observed in the H295R cell assay with a rank order potency consistent with assays conducted using purified protein (cf. Fig. 6, Table 1 and Table S1). The Gaussian distributions observed for 11-deoxycorticosterone and corticosterone in cells exposed to abiraterone confirm that it inhibits both CYP17A1 and CYP21A2 at concentration ranges of 2.5–10 nM and 30–3000 nM, respectively. Similar effects have been detected for **1** and particularly **2**, with possible inhibition of CYP21A2 at concentrations greater than 1000 nM. Weak inhibition of CYP21A2 is consistent with the low affinity for purified CYP21A2 based on the UV-VIS spectral shift assay.

This shows that compounds **1** and **2** are more selective for CYP17A1 *vs.* other cytochrome P450 enzymes than abiraterone. A further characterisation of the compounds in different cancer cell lines^33^ and animal models of human prostate cancer^34^ will address the clinical potential of compounds **1** and **2**. Mineralocorticoid excess may still be observed because these new compounds still inhibit both the CYP17A1-mediated hydroxylase and the 17,20-lyase reaction with similar IC_50_ values (cf. Table 1) although a recent patent application has suggested that compounds similar to **1** inhibit the lyase step^35^. Rafferty *et al.* have demonstrated a strategy for generating lyase-selective inhibitors by replacing nitrogen heterocycles with strong coordination to the haem with heterocycles that interact more weakly^11^. Thus, the similar IC_50_ values of compound **1** and **2** for inhibition of hydroxylation compared to 17,20-lyase activity may be due to the presence of a strong haem-coordinating group such as the pyridine. VT-464, with IC_50_ values of 670 and 69 nM for the hydroxylase and the lyase steps, respectively, coordinates the haem iron by 1,2,3-triazole ring^11^, which is a weaker binder than a pyridine ring (cf. Fig. 3). Thus, modifications of **1** and **2** replacing the pyridine ring with less pronounced haem-interacting heterocycles, *e.g.* an isoxazole or a tetrazole may generate compounds with increased selectivity for the 17,20-lyase reaction vs. the hydroxylase step.

To summarize, we combined DFT calculations, docking with ChemScore, ligand-strain determination, and QM/MM optimizations to identify non-steroidal CYP17A1 inhibitors. Compounds **1** and **2** bound CYP17A1 tightly and effectively inhibited both hydroxylation and the 17,20-lyase reaction in a purified enzyme system. Strong coordination of compounds **1** and **2** to the haem iron is likely responsible for inhibition of both reactions. These compounds do not bind selected drug-metabolizing cytochrome P450 enzymes or the steroidogenic CYP21A2, suggesting a reduced risk for undesirable side effects, especially on the corticosteroid production, consistent with our *in vitro* data. Taken together, these data recommend compounds **1** and **2** as promising tools for the continued development of new drugs against prostate cancer.

## Methods

ZINC^18^ and eMolecules^19^ databases were used as reservoirs of commercially available compounds for virtual screening. The hits tested were purchased from Chembridge, Chemical Division, Enamine, InterBioScience, Life Chemicals, Maybridge and Vitas M Labs. Abiraterone was bought from Tokyo Chemical Industry.

### Binding energy calculations on small nitrogen-containing aromatic rings

Density functional theory (DFT) method was used to calculate the binding energy of the most common N-containing heterocycles to a simplified haem modelled as a ferric (Fe^III^) ion embedded in a porphyrin ring with an axial methyl mercaptide group. The latter mimics the cysteine residue that coordinates the iron in cytochrome P450 enzymes (see Figure S1, Supplementary Information). Calculations were carried out at the B3LYP level of theory with unrestricted formalism for open-shell systems in the doublet spin state^36^,^37^,^38^. Geometry optimizations were performed in the gas phase using the 6–31 G(d) basis set^39^ for all atoms except iron, for which the double-ξ basis set of Schäfer *et al.*^40^ enhanced with a p function was applied. Solvation effect calculations were carried out at the same level of theory with the continuum conductor-like screening model (COSMO)^41^ using an effective dielectric constant of 4. The final binding energy (ΔΔ*E*, see equation 1) was calculated according to the process outlined in Fig. 3 as the difference of the interaction between the heterocycle alone and in complexes with water and with the haem, respectively. All the calculations were performed using the software package TURBOMOLE^42^.


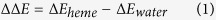



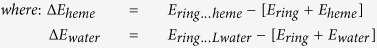


### Filtering and clustering strategies for ZINC and eMolecules databases

The ZINC database^18^ was screened for compounds containing each of the best iron-interacting heterocycles in their structures and with a molecular weight lower than 500 g·mol^−1^. The queries for eMolecules were restricted to compounds containing only imidazole and pyridine rings, as they are the best binders to the haem-iron according to our DFT calculations. Several filtering processes using the software CANVAS^43^ were applied to decrease the number of molecules to screen. First, a diversity-based selection was made on the two libraries and then all the hits with molecular weight higher than 500 g·mol^−1^ and > 35 heavy atoms were discarded. Finally, binary fingerprints of the remaining compounds were determined, and the so-called “leader-follower” clustering was performed on the matrix of the pair similarities using the Tanimoto metric (cluster radius of 0.8). We continued working only with the leaders, as they should summarize the features present in each cluster.

### Preparation of protein and compounds for docking

The X-ray structure of CYP17A1 in complex with the inhibitor TOK-001 (PDB ID 3SWZ, resolution nof 2.40 Å) was used^8^. The protein was prepared for docking using the Protein Preparation Wizard suite implemented in MAESTRO^44^. Hydrogen atoms, bond orders, and missing residues were added to the initial coordinates, while water molecules were removed from the binding pocket. The structure was protonated according to pH = 7.0 and the positions of the hydrogen atoms optimized using the OPLS-2005 force field.

Ligands were energy minimized in the MacroModel suite^45^ of MAESTRO (OPLS-2005 force filed, default settings). Protonation states and possible tautomers were then generated ant pH = 7.0 ± 2.0 using the Epik programme^46^.

### Docking

Docking was performed with GOLD (Genetic Optimization for Ligand Docking) version 5.2^23^ with the haem-tailored ChemScore function developed by Kirton *et al.* as scoring function^24^. The protein was kept rigid, while single bonds of the ligands were treated as rotatable. The docking radius was set to 15 Å around the center of mass of the co-crystallized ligand and 50 independent docking runs were performed. To generate a different binding pose for compound **2**, the distance between the haem iron and the pyridine nitrogen atom was constrained between 1.5 and 3.0 Å, following the default protocol in GOLD. During the screening of the compounds from ZINC and eMolecules databases, the genetic algorithm was allowed to terminate if the RMSD of the top three solutions for each hit differed by less than 1.5 Å.

The protocol was evaluated in terms of atom-positional root-mean-square-deviation (RMSD) of the obtained poses with respect of the co-crystallized ligand (RMSD of 0.82 Å).

### Ligand strain calculations

All the calculations were performed in MacroModel^45^ using the OPLS-2005 force field with default settings. To compare the energy of the docked poses with the minimum energy conformations, each docked pose from GOLD was adjusted to the OPLS-2005 force filed in MAESTRO. To do this, a constrained minimization of the heavy atoms was performed with a force constant of 100 kJ·(mol^−1^-Å^−2^). Free movement of the heavy atoms within ±0.3 Å was facilitated by the use of a flat-bottomed potential, whereas hydrogen atoms were kept free. The global minimum for each hit was obtained from a Monte Carlo conformational search.

### QM/MM refinement of the systems

The QSite programme^47^ was used to set up and run the calculations on the hits that survived the ligand strain filter. The ligand, the haem without the carboxylic groups, and the full Cys442 residue interacting with the iron atom were treated quantum mechanically using a spin unrestricted (doublet) DFT-B3LYP level of theory with the LACVP* basis set. The molecular mechanics (MM) part was represented by the rest of the protein, which was allowed to relax following the default protocol.

### Protein expression and purification

Human CYP17A1 bearing a truncation of the N-terminal helix and a 4x histidine tag on the C-terminus was expressed and purified as previously reported^48^.

### Ligand binding assays

Ligand binding was measured based on a change in UV-VIS absorbance (ΔA) upon ligand addition. Purified recombinant human cytochrome P450 enzymes (100 nM) were titrated with ligands dissolved in DMSO in 5 cm path length cuvettes. CYP17A1 and CYP21A2 were prepared in 50 mM Tris-HCl, pH 7.4, 500 mM NaCl, 20% (v/v) glycerol, and 100 mM glycine. The buffer used to prepare CYP21A2 also contained 50 mM DTT. CYP3A4 and CYP2D6 were prepared in 50 mM potassium phosphate, pH 7.4, 250 mM NaCl, 20% (v/v) glycerol and 1 mM EDTA. In each case, heteroatom coordination to the haem iron yields an increase in absorbance at ~420 nm and a corresponding decrease at ~390 nm. The apparent binding constant (*K*_d_) and the maximum spectral change (ΔA_max_) were calculated from a nonlinear least-squares regression fit to the tight binding equation (2) using GraphPad Prism 6.03.


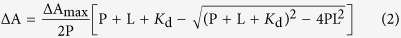


where P and L are the total CYP17A1 and ligand concentrations, respectively.

### Hydroxylase and 17,20-lyase inhibition with purified CYP17A1

Selected compounds were assessed for inhibition of progesterone 17α-hydroxylase and 17α-hydroxypregnenolone 17,20-lyase activity using a purified protein system. Purified cytochrome P450 17A1 (50 nmol) was incubated with truncated human cytochrome P450 reductase^48^ and rat cytochrome *b*_5_ in a 1:4:0 or 1:4:4 ratio for hydroxylase and 17,20-lyase reactions, respectively. The 17α-hydroxylase inhibition was evaluated using 10 μM progesterone as substrate and inhibitor concentrations of 0–300 nM for abiraterone, 0–5000 nM for **1,** and 0–1000 nM for **2**. Reactions were initiated upon addition of 25 mM NADPH as the electron source. The hydroxylated product was quantified after HPLC using an UV detector and analyzed as previously published^8^. The 17,20-lyase assay was performed similarly substituting 1.2 μM of 17α-hydroxypregnenolone as the substrate and inhibitor concentrations of 0–500 nM. The dehydroepiandrosterone product was quantitated using a coupled GC/MS method^48^.

### H295R cell assay

The H295R steroid hormone synthesis assay was performed according to the OECD validation guideline^49^. Cells were cultured in DMEM/F12 media supplemented with 1% ITS-premix and 2.5% Nu-serum at 37 °C with a 5% CO_2_ atmosphere. The cells were only used for experiments between passage 4–12°. During exposure experiments, cells were grown in 24 well plates with a density of 3 × 10^5^ cells/mL. Cells were allowed to settle for 24 hours after which the medium was changed and abiraterone (0.0001–10 μM), compound **1** (0.0001–10 μM), or **2** (0.001–60 μM with precipitation observed at the highest concentration) added. To avoid interference from low levels of steroid hormones present in the Nu-serum, the exposure experiment was conducted with serum-free media. Each compound was tested at a minimum of seven concentration levels using three or more replicates and the experiment was repeated on two different days (n = 6–15). On each test plate a solvent control (SC) (medium with 0.1% DMSO) was included in triplicate and the maximal concentration of DMSO in the cell medium was 0.1%^49^. After 48 hours of incubation in presence of the test compounds, 950 μL of the medium was carefully removed and internal standards (50 μl of 0.1 μg/μl solution containing deuterated steroid analogues) were added. Samples were stored at −20 °C for later analysis.

Cell viability was confirmed with the resazurin assay, as described by Nielsen *et al.*^50^. All tested concentrations confirmed viable cells with the exception of cells exposed to 10 μM of abiraterone.

Steroid extraction and clean-up were performed by double protein precipitation. First, 900 μL of cold acetonitrile were added to the samples, which were then vortexed and centrifuged at ~9500 × g for 10 minutes. Second, 900 μL cold methanol was added to the supernatant and the mixture was vortexed and centrifuged at 1500 × g for 10 minutes. Finally, the supernatant was collected and concentrated to ~1 mL under a gentle stream of nitrogen at 60 °C.

### Analysis of the H295R samples using LC and MS

For on-line solid phase extraction (SPE) of steroids, a total of 100 μL was injected on a binary 1290 Agilent Infinity Series system and a binary 1100 Agilent HPLC pump were used in combination (Agilent Technologies, Palo Alto, CA, USA). For online clean-up, a C_18_ enrichment column (μbondapak^®^ C_18_, 3.9 × 20 mm, 10 μm, Waters) was used. The enrichment column was connected to the autosampler through the TTC switching valve (two positions, 6 ports). Between the autosampler and the TTC switching valve a 0.3 μm in-line filter (1290 infinity in-line filter, Agilent) was installed. Separation of steroid hormones was performed using a C_18_ analytical column (Kinetex, 2.6 μm C_18_ 100 Å, 75 × 2.1 mm, Phenomenex, USA). An isocratic flow of 1 mL/min H_2_O:methanol:formic acid at 90:10:0.1 (v/v/v) was generated by the 1100 pump which was connected to the autosampler. The 1290 pump performed a gradient elution with a flow rate of 0.3 mL which was connected to the TTC switching valve. Mobile phase A and B were composed of H_2_O with 0.1% formic acid (v/v) and pure methanol, respectively. The elution gradient was maintained at 10% B for the first 2 min, changed to 10.0–30.0% from 2.0 to 2.2 min, 30.0–60.0% from 2.2 to 8.0 min, maintained at 60.0% from 8.0 to 10.0 min, increased to 60.0–85.0% from 10.0 to 12.30 min, 85.0–99.5% from 12.3 to 12.5 min and held at 99.5% from 12.5 to 14.8 min, before re-equilibrating the column until 16 minutes^51^.

For detection, an AB SCIEX 4500 QTRAP mass spectrometer (Applied Biosystems, Foster City, CA, USA) equipped with an atmospheric pressure chemical ionization (APCI) Turbo V source was used. Multiple reaction monitoring (MRM) was performed in positive mode during the analysis with a targeted scan time of 0.8 sec. Nitrogen was applied as curtain, collision, and ion source gases. The obtained ions with their retention times and molar masses are listed in Table S3.

LC and MS optimizations were conducted using Analyst v. 1.6.2 software package (AB SCIEX) and obtained chromatographic peaks and quantification were processed in MultiQuant v. 3.0 software (AB SCIEX). Calculations and graphics were performed using Microsoft Office Excel 2010 and GraphPad Prism v. 6.03 (GraphPad Software, San Diego, CA, USA).

## Additional Information

**How to cite this article**: Bonomo, S. *et al.* Promising Tools in Prostate Cancer Research: Selective Non-Steroidal Cytochrome P450 17A1 Inhibitors. *Sci. Rep.* **6**, 29468; doi: 10.1038/srep29468 (2016).

## Supplementary Material

Supplementary Information

## Figures and Tables

**Table 1:** Biological characterization of compound **1**, **2** and abiraterone in the isolated human CYP17A, in the H295R cell system and their selectivity profiles against other CYPs.

Compound	*K*_d_ [nM]^a^	Purified CYP17A1		H295R system		Selectivity		
		IC_50_ [nM]^c^		IC_50_ [nM]^d^		UV-VIS spectral shift		
		hydroxylase	lyase	hydroxylase^e^	lyase^f^	CYP 3A4	CYP 2D6	CYP 21A2
**1**	≪100^b^	230 ± 20	500 ± 90	830 ± 80	94 ± 30	−	−	−
**2**	≪100^b^	130 ± 10	110 ± 20	52 ± 4	7.4 ± 0.1	−	−	−
**abiraterone**	≪100^b^	92 ± 4	36 ± 2	9.4 ± 0.3	1.7 ± 0.1	+	+	+

^a^Binding measurements with UV-VIS. Mean value over 2 measurements ± standard error.

^b^The measurement suffers of intrinsic inaccuracy because of the *K*_d_ value smaller than the lowest protein concentration at which the titration can be performed with acceptable signal: noise (100 nM).

^c^Inhibition measurements in recombinant CYP17A1. Mean value over 3 measurements ± standard error. Assays based on the conversion of PRO to 17-OH-PRO and 17-OH-PRE into DHEA for hydroxylase and lyase, respectively.

^d^Inhibition in H295R cells. Mean value over 6 to 15 measurements ± standard error.

^e^Based on the conversion of PRE into 17-OH-PRE.

^f^Based on the conversion of 17-OH-PRE into DHEA. Abbreviations are defined in Fig. 1.
