# Supplementary material for: Promising Tools in Prostate Cancer Research: Selective Non-Steroidal Cytochrome P450 17A1 Inhibitors
Source: Sci Rep. 2016 Jul 12;6:29468. doi: 10.1038/srep29468 (PMC4942611; doi:10.1038/srep29468)
Supplement: Supplementary Information [file srep29468-s1.pdf]

Supplementary Information

Promising Tools in Prostate Cancer Research –Selective Non-Steroidal Cytochrome P450 17A1 Inhibitors

Silvia Bonomo[1], Cecilie H. Hansen[2], Elyse M. Petrunak[3], Emily E. Scott[3], Bjarne Styrishave[2], Flemming Steen Jørgensen[1] and Lars Olsen[1,]*

[1]Department of Drug Design and Pharmacology, University of Copenhagen, Universitetsparken 2, DK-2100, Copenhagen Ø, Denmark.

[2]Department of Pharmacy, University of Copenhagen, Universitetsparken 2, DK-2100 Copenhagen Ø, Denmark.

[3]Department of Medicinal Chemistry, The University of Kansas, 1251 Wescoe Hall Dr., Lawrence, KS, 66045 USA.

Contents

Figure S1: Training set of compounds used to select the best ChemScore cutoff for screening the docking results.

Figure S2: 2D representation of the simplified heme model and the heterocycles used for the DFT calculations.

Figure S3: Virtual hits from ZINC and eMolecules databases.

Figure S4: Absorbance difference vs. ligand concentration plots for compounds **1-5**.

Figure S5: Structures of compound 2 and its commercially available analogues tested for their binding properties to CYP17A1.

Table S1: Biological characterization of compound **3-7** with the purified, human CYP17A1.

Table S2: Complete biological characterization of compound **1**, **2** and abiraterone on the production of steroidal hormones in the H295R cell line.

Table S3: Ionization fragments, retention times and molar masses for on-line clean-up and analysis of steroids from the H295R assay.

Appendix 1. Purity data for compound **1** provided by MayBridge LTD vendor

Appendix 2. Purity data for compound **2** provided by the ChemBridge vendor.

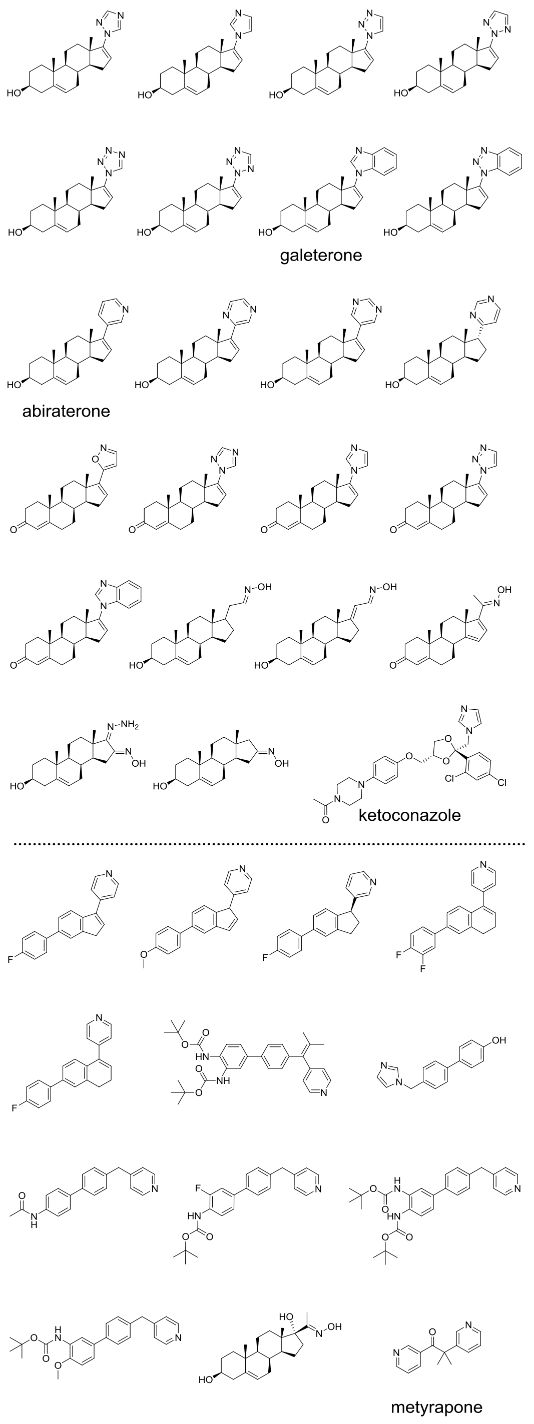

Figure S1. Training set of compounds used to select the best ChemScore cutoff for screening the docking results. The top panel contains inhibitor compounds (IC_{50} < 5000 nM), whereas the non-inhibitors (IC_{50} > 5000 nM) are shown in the lower panel[1, 2, 3].

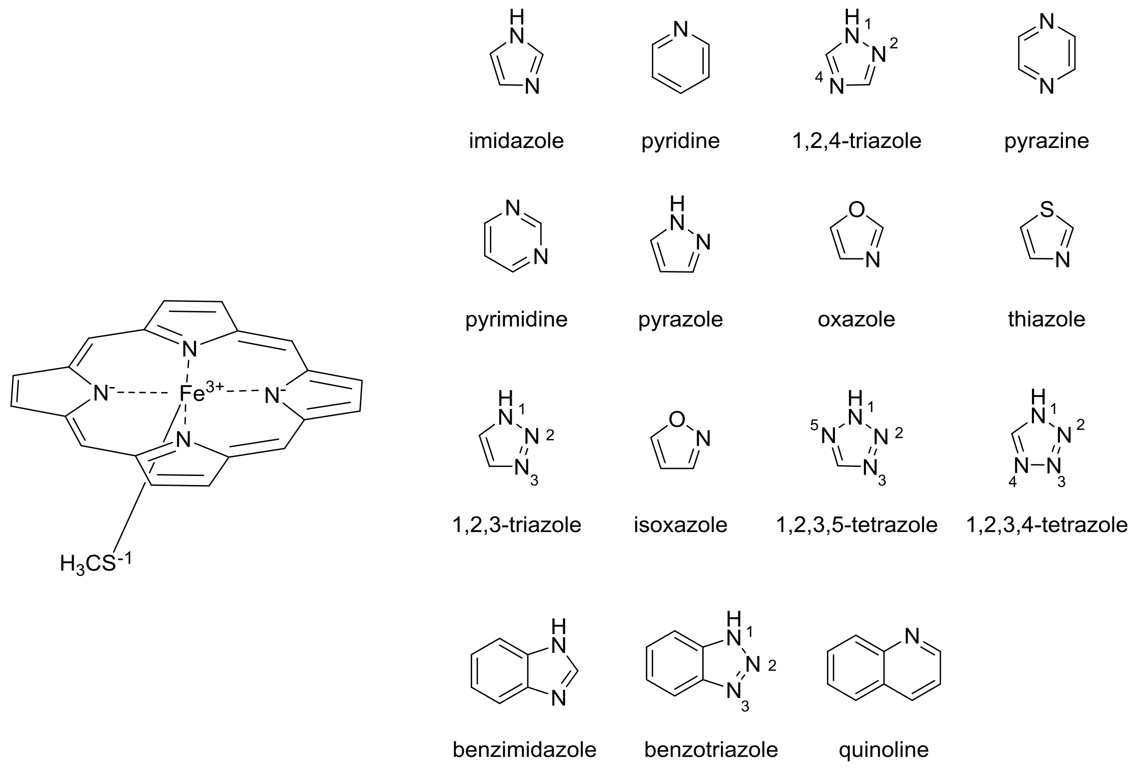

Figure S2. 2D representation of the simplified heme model (**a**) and the heterocycles (**b**) used for the DFT calculations. The order in which the structures are reported resembles the rank obtained by the DFT calculations and shown in Figure 2 of the main paper.

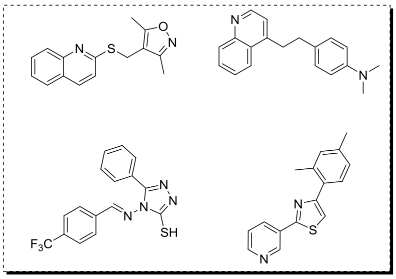

virtual hits from ZINC database

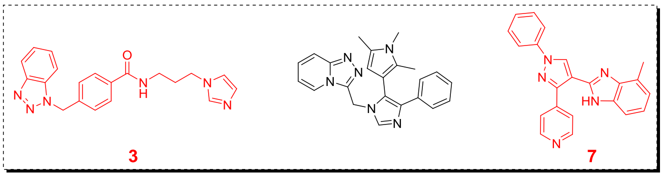

virtual hits from the imidazole-based eMolecules database

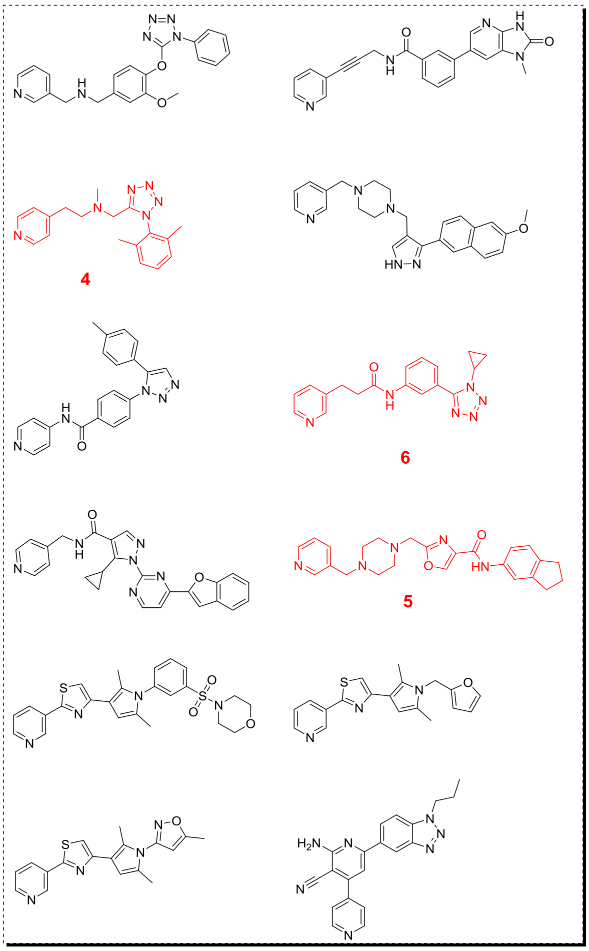

virtual hits from the pyridine-based eMolecules database

Figure S3. Virtual hits from ZINC and eMolecules databases. These compounds were tested for their ability to coordinate the heme iron by recording their UV-VIS spectrum in presence of CYP17A1. Compounds colored in red gave a type II shift of the UV-VIS spectrum, but were not able to inhibit the enzyme activity.

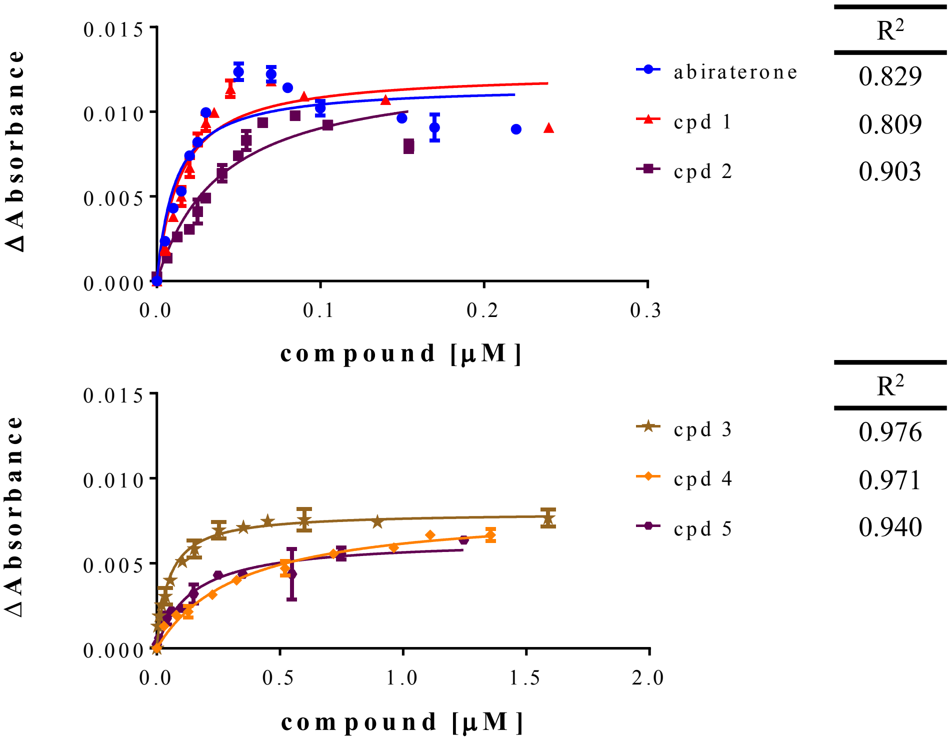

Figure S4. Absorbance difference against ligand concentration plots for compounds **1-5** and abiraterone. R^2 values for each data fitting to the binding equation are reported.

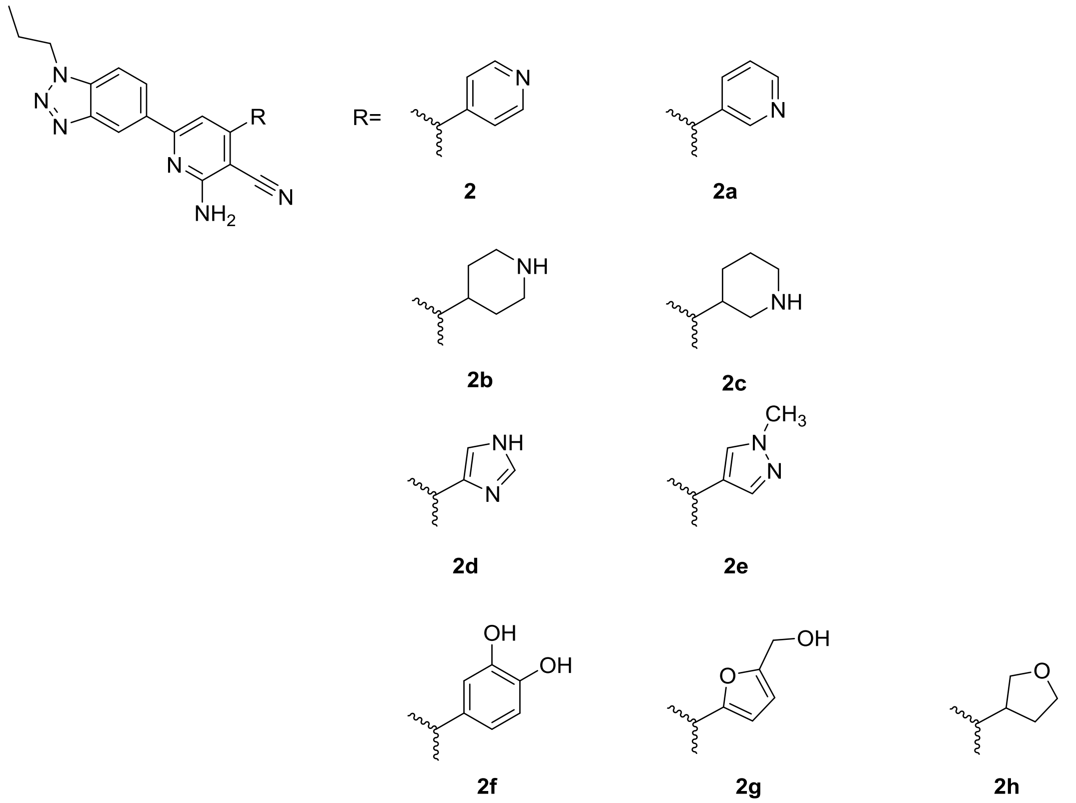

Figure S5. Structures of compound **2** and its commercially available analogues tested for their binding properties to CYP17A1.

Table S1. Biological characterization of compound **3-7** with the purified, human CYP17A1.

**Compound**	K_d **[nM]**[a]	**IC$_{50}$ [nM]**[b]	
		hydroxylase	lyase
**3**	400±40	>10000	ND[e]
**4**	<<100	>10000	ND[e]
**5**	150±20[d]	>10000	ND[e]
**6**	ND[e]	>10000	ND[e]
**7**	ND[e]	>10000	ND[e]

[a]Mean value over 2 measurements±standard error. [b]Mean value over 3 measurements±standard error. Assays based on the conversion of progesterone in 17α-hydroxyprogesterone and 17α-hydroxypregnenolone into DHEA for hydroxylase and lyase, respectively. [c]The measurement suffers of intrinsic inaccuracy because of the K_d value smaller than the lowest protein concentration at which the titration can be performed with acceptable signal:noise (100 nM). [d]Calculated using the highest absorbance data. [e]ND: not determined.

Table S2. Complete biological characterization of the effects of compound **1**, **2** and abiraterone on the production of steroidal hormones in the H295R cell line.

	compound 1		compound 2		abiraterone	
	IC_{50} [nM][a]	P-value	IC_{50} [nM][a]	P-value	IC_{50} [nM][a]	P-value
**Progestagens**						
PREG	390±70	0.1024	15±3	0.156	5.9±0.5	0.010
	500±70[b]	0.0749	19±4[b]	0.0632	7.3±0.3[b]	0.0028
PROG	ND[c]	ND[c]	ND[c]	ND[c]	181±5	<0.0001
	146±7[b]	0.0086	32±4[b]	0.0518	1.0±0.1[b]	0.0014
17OH-PREG	830±80	0.0153	52±4	0.0038	9.7±0.3	<0.0001
17OH-PROG	800±200	0.1705	20±1	0.0008	6.3±0.2	<0.0001
**Corticosteroids**						
11deoxy-COS	310±60	0.1633	23.8±0.9	0.0002	2.6±0.5[d]	0.0304
	146±4[b]	0.0144			240±20[e]	
COS	ND[c]	ND[c]	36±4	0.021	2.9±0.5[d]	0.0179
	56±1[b]	0.0285				
11deoxy-COR	480±10	<0.0001	54±5	0.017	5.3±0.1	<0.0001
COR	1200±100	0.0135	36±4	0.028	5.0±0.4	0.0092
CORNE	700±100	0.0777	23±1	0.0005	2.5±0.1	<0.0001
**Androgens**						
DHEA	94±30	0.0001	7.4±0.1	<0.0001	1.8±0.1	<0.0001
AN	68±2	<0.0001	4.3±0.1	<0.0001	0.5±0.0	<0.0001
TS	52±2	<0.0001	3.3±0.2	0.0006	0.2±0.0	0.0024
**Estrogens**						
E1	220±10	0.0004	19±3	0.0597	0.8±0.1	0.0053
β-E2	235±6	0.0001	50±10	0.146	408±60	0.0953

[a]Mean value over 6 to 15 measurements±standard error. [b]EC_{200}±standard error and relative P-value. [c]ND: not determined. [d]Value calculate on low concentrations of abiraterone (first part of the Guassian-like curve). [e]Value calculated using high concentrations of abiraterone (second part of the Guassian-like curve).

Table S3. Ionization fragments, retention times and molar masses for on-line clean-up and analysis of steroids from the H295R assay.

Steroid[a]	IS[b]	$C_xH_yO_z$[c]	M[d]	Precursor Ion	Quantifier Ion	Qualifier Ion	RT[e]
AN	AN-d7	$C_{19}H_{26}O_2$	286.4	287.1	96.9	108.9	9.29
ADIOL	DHT-d3	$C_{19}H_{30}O_2$	290.4	255.2[e]	159.0	145.0	11.19
DHEA	DHEA-d6	$C_{19}H_{28}O_2$	288.4	271.2[f]	213.1	159.0	10.29
TS	TS-d3	$C_{19}H_{28}O_2$	288.4	289.1	96.9	108.9	9.84
ALDO	COR-d4	$C_{21}H_{28}O_5$	360.4	361.1	315.0	325.0	6.91
COR	COR-d4	$C_{21}H_{30}O_5$	362.4	363.1	121.0	327.2	7.62
11-deoxyCOR	11-deoxyCOR-d5	$C_{21}H_{30}O_4$	346.4	347.0	108.9	96.9	8.74
COS	COS-d8	$C_{21}H_{30}O_4$	346.4	347.0	121.0	163.1	8.56
11-deoxyCOS	11-deoxyCOS-d8	$C_{21}H_{30}O_3$	330.4	331.1	108.9	96.9	9.66
CORNE	COR-d4	$C_{21}H_{28}O_5$	360.4	361.1	163.1	121.0	7.26
E1	E1-d4	$C_{18}H_{22}O_2$	270.4	271.3	132.9	197.1	9.46
βE2	βE2-d5	$C_{18}H_{24}O_2$	272.4	255.2[f]	159.0	132.9	9.58
PREG	PREG-d4	$C_{21}H_{32}O_2$	316.5	299.1†	281.1	159.0	13.10
17-OHPREG	DHEA-d6	$C_{21}H_{32}O_3$	332.4	297.1[e]	279.1	159.0	10.49
PROG	PROG-d9	$C_{21}H_{30}O_2$	314.5	315.2	108.9	279.1	12.20
17-OHPROG	DHEA-d6	$C_{21}H_{30}O_3$	330.4	331.1	108.9	313.0	10.17

[a]See Figure 1 of the main paper for abbreviations; [b]internal standard; [c]molecular formula; [d]molar mass; [e]M+1 -18; [f]M+1 -2 x 18.

Appendix 1. Purity data for compound **1** provided by MayBridge LTD vendor.

131917

Analysis Number	**131917**	*Injection Volume*	**10.0**
Compound Code	**ML00080**	*Mol.Weight*	**347**
Batch/Book No		*Submitter*	**QC**
Project Code	**QC-SCR**	*Sample Tray*	**CStk1_01**
Program File	**Test_1G**	*Sample Position*	**40**
Quantif'n Method	**High_5 params**	*Analyst*	**SMB**
Data Collected	**08/02/16 14:21**	*PDA Wavelength*	**215**

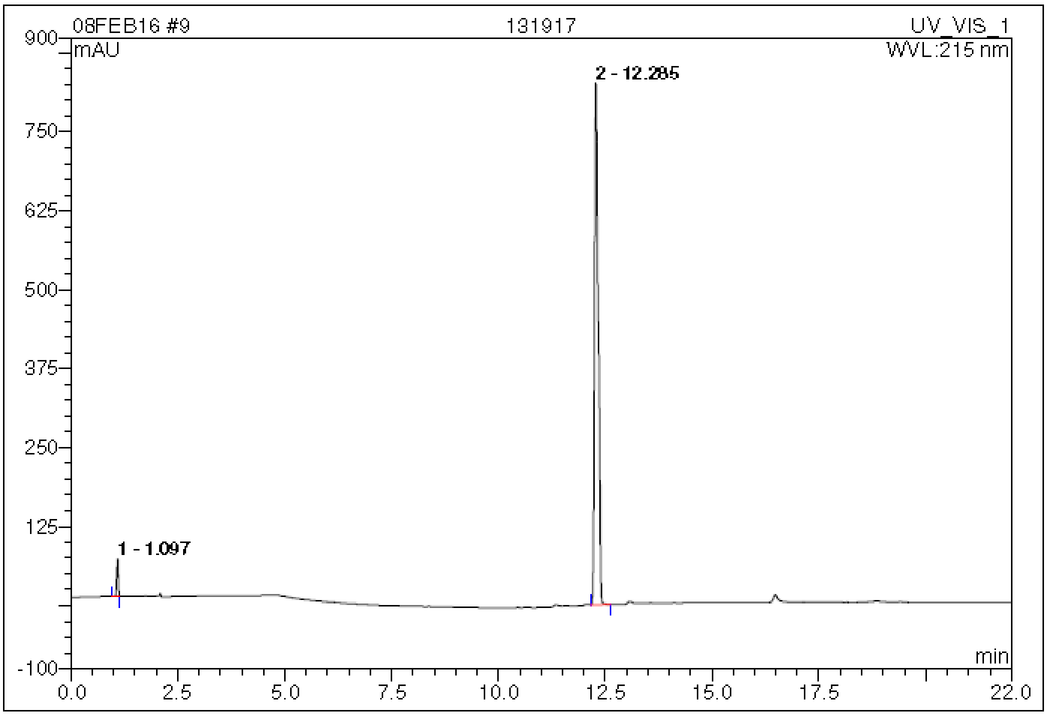

No.	Ret.Time min	Height mAU	Area mAU*sec	Rel.Area %	
1	1.10	59.607	157.324	3.08	
2	12.29	825.831	4957.983	96.92	
**Total:**		885.438	5115.307	100.00	

HPLC data

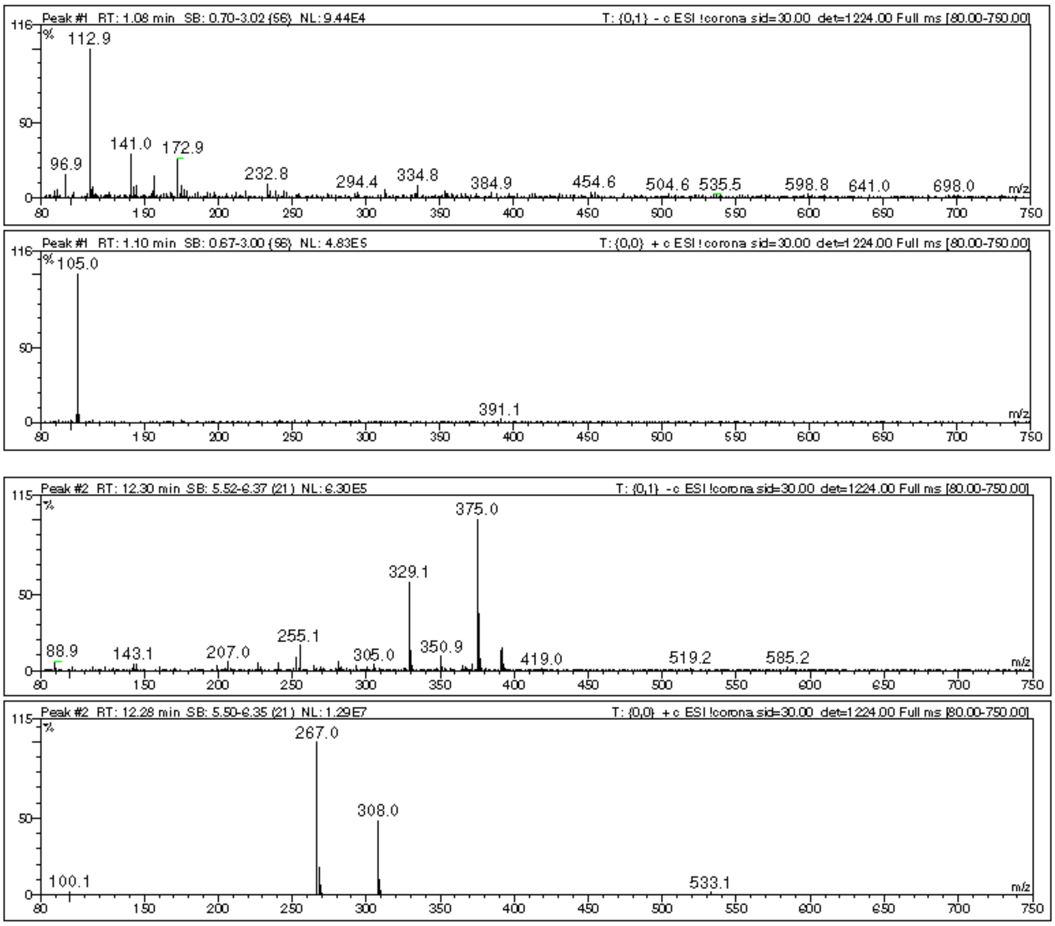
Peak #1 RT: 1.08 min SB: 0.70-3.02 (56) NL: 9.44E4
T: {0,1} - c ESI !corona sid=30.00 det=1224.00 Full ms [80.00-750.00]
112.9
141.0
172.9
96.9
232.8
294.4
334.8
384.9
454.6
504.6
535.5
598.8
641.0
698.0
Peak #1 RT: 1.10 min SB: 0.67-3.00 (56) NL: 4.83E5
T: {0,0} + c ESI !corona sid=30.00 det=1224.00 Full ms [80.00-750.00]
105.0
391.1
Peak #2 RT: 12.30 min SB: 5.52-6.37 (21) NL: 6.30E5
T: {0,1} - c ESI !corona sid=30.00 det=1224.00 Full ms [80.00-750.00]
375.0
329.1
255.1
88.9
143.1
207.0
305.0
350.9
419.0
519.2
585.2
Peak #2 RT: 12.28 min SB: 5.50-6.35 (21) NL: 1.29E7
T: {0,0} + c ESI !corona sid=30.00 det=1224.00 Full ms [80.00-750.00]
267.0
308.0
100.1
533.1
m/z

MS data

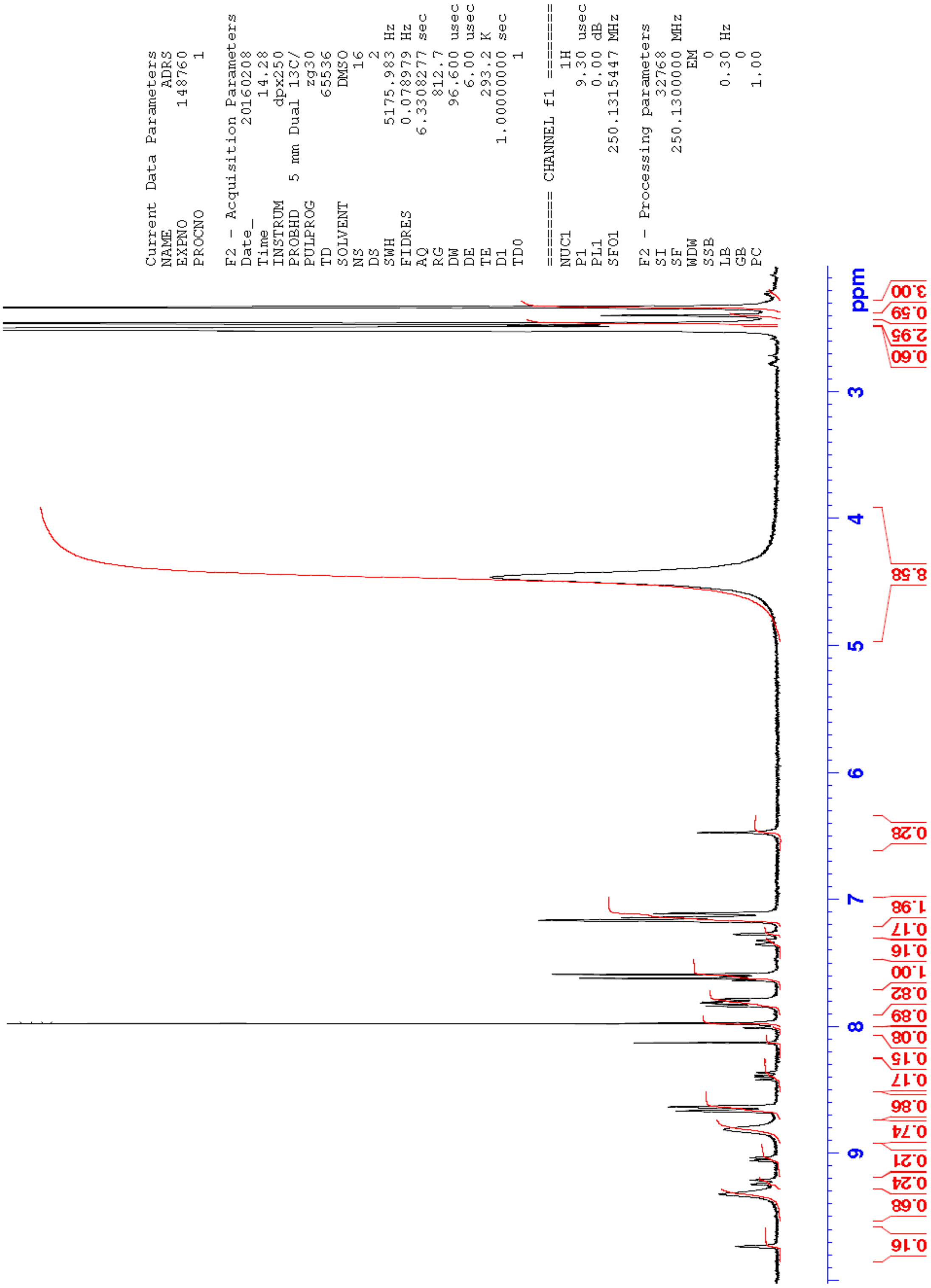

^{1}H NMR spectrum

According to the NMR data, the sample is a mixture of hydrobromide salt (87 %) and free base (13%) of **1.**

Appendix 2. Purity data for compound **2** provided by the ChemBridge vendor.

FC94559331

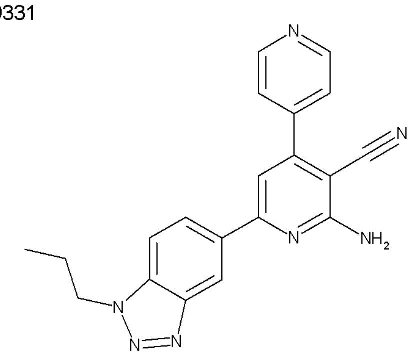

ID	53137298	355.4054	$C_{20}H_{17}N_7$

```
Data File R:\HPLC\AUTO\FC945593\1GD-1501.D
Sample Name: FC945593P1-G-04
Instrument 1 11/10/2011 17:26:30
PMP1, Solvent A         : 0.1%TFA in Acn/H2O
(2.5:97.5)
PMP1, Solvent B         : 0.1% TFA in AcN
PMP1, Solvent C         : 0.1%FA in ACN/H2O
(2.5:97.5)
PMP1, Solvent D         : 0.1%FA in ACN
Ionization mode             :    APCI Positive

Signal 1: ADC1 A, ELSD
Peak RetTime Type  Width     Area      Height
Area
  #   [min]        [min]   [mV*s]      [mV]        %
----|-------|----|-------|----------|----------|-----
---|
   1   1.247 MM    0.0411  197.33905   79.97787
100.0000
Totals :                   197.33905   79.97787


Signal 2: DAD1 A, Sig=300,200 Ref=off
Peak RetTime Type  Width     Area      Height
Area
  #   [min]        [min]   [mAU*s]     [mAU]       %
----|-------|----|-------|----------|----------|-----
---|
   1   1.192 MM    0.0412 3146.31299 1273.87756
100.0000
Totals :                  3146.31299 1273.87756


Signal 3: MSD1 TIC, MS File
Peak RetTime Type  Width     Area      Height
Area
  #   [min]        [min]                           %
----|-------|----|-------|----------|----------|-----
---|
   1   1.318 MM    0.0825 8.56650e6  1.73153e6
100.0000
Totals :                  8.56650e6  1.73153e6
```

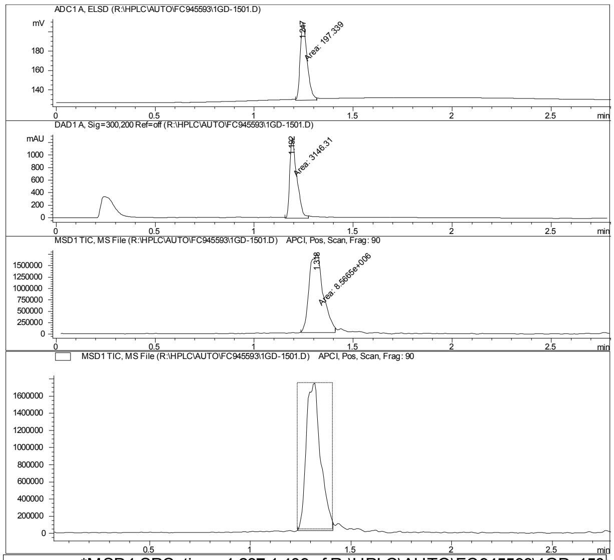

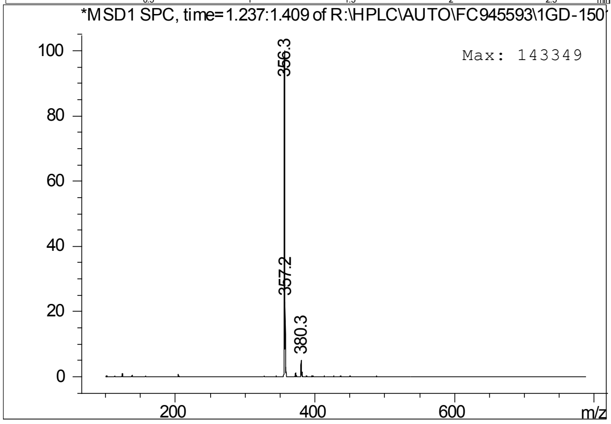

References

1. Pinto-Bazurco Mendieta MAE, Negri M, Jagusch C, Müller-Vieira U, Lauterbach T, Hartmann RW. Synthesis, Biological Evaluation, and Molecular Modeling of Abiraterone Analogues: Novel CYP17 Inhibitors for the Treatment of Prostate Cancer. *J. Med. Chem.* **51**, 5009-5018 (2008).

2. Hu Q, Jagusch C, Hille UE, Haupenthal Jr, Hartmann RW. Replacement of Imidazolyl by Pyridyl in Biphenylmethylenes Results in Selective CYP17 and Dual CYP17/CYP11B1 Inhibitors for the Treatment of Prostate Cancer. *J. Med. Chem.* **53**, 5749-5758 (2010).

3. Hu Q, Yin L, Jagusch C, Hille UE, Hartmann RW. Isopropylidene Substitution Increases Activity and Selectivity of Biphenylmethylene 4-Pyridine Type CYP17 Inhibitors. *J. Med. Chem.* **53**, 5049-5053 (2010).
